# Diagnostic accuracy of deep learning models for colorectal cancer detection in histopathological whole-slide images: a systematic review and diagnostic test accuracy meta-analysis

**DOI:** 10.3389/fonc.2026.1885546

**Published:** 2026-08-17

**Authors:** Xiang Yong, Zhen Li, Qiang Wu, Huaiyuan Hu

**Affiliations:** 1 Department of Pathology, Wanbei Coal-Electricity Group General Hospital, Suzhou, Anhui, China; 2 Suzhou City Key Laboratory of Tumor Pathology, Suzhou, Anhui, China; 3 Department of Pathology, The Affiliated Changsha Hospital of Xiangya School of Medicine, Central South University/The First Hospital of Changsha, Changsha, Hunan, China; 4 Department of Pathology, The Second Affiliated Hospital of Anhui Medical University, Hefei, Anhui, China

**Keywords:** colorectal cancer, deep learning, diagnostic test accuracy, digital pathology, histopathology, meta-analysis, whole-slide imaging

## Abstract

**Background:**

Deep learning systems are increasingly used for colorectal cancer detection in digital histopathology, but studies often report heterogeneous endpoints, patch-level metrics, or non-thresholded area under the curve (AUC) values. We estimated the diagnostic accuracy of clinically interpretable slide-level or patient-level models.

**Methods:**

We searched PubMed, Embase, Scopus, Web of Science Core Collection, and IEEE Xplore for deep learning studies of human colorectal histopathology or whole-slide images. Eligible studies provided extractable or reconstructable slide-level or patient-level true-positive (TP), false-positive (FP), false-negative (FN), and true-negative (TN) values for colorectal cancer, colorectal adenocarcinoma, or closely aligned malignant colorectal histopathology detection. Risk of bias was assessed using QUADAS-2 with AI-pathology-specific considerations. The strict primary synthesis excluded endpoint-caveat cohorts. Summary sensitivity and specificity were estimated using a bivariate random-effects Reitsma model. A second reviewer verified eligibility, 2 x 2 tables, and QUADAS-2 judgments.

**Results:**

The search identified 4,687 records; 2,307 unique records were screened. Three source studies contributing 14 validation cohorts met the inclusion criteria for the strict primary synthesis; five additional endpoint-caveat studies contributing seven cohorts were retained only for expanded and sensitivity analyses. Primary cohorts included 5,402 TP, 439 FP, 68 FN, and 3,596 TN. The exploratory bivariate model, which treated cohorts as independent observations, estimated a sensitivity of 0.982 (95% CI, 0.976–0.986), a specificity of 0.959 (95% CI, 0.922–0.979), and an AUC of 0.979. Wang (2021) contributed 12 of 14 cohorts; within-study correlation could therefore make these confidence intervals overly precise. Sensitivity was stable across scenarios, whereas crude specificity varied with endpoint definition, analysis unit, and large true-negative denominators.

**Conclusion:**

Available cohort-level evidence suggests high sensitivity and strong overall discrimination for colorectal cancer detection on histopathological whole-slide images. However, the exploratory estimates are dominated by one development program and should not be interpreted as a mature, independently replicated, multisource evidence base. Specificity remains vulnerable to endpoint heterogeneity and slide-level calling rules. Current evidence supports the use of deep learning as an adjunct for prescreening, triage, or quality control, rather than as a standalone diagnostic replacement.

## Introduction

Colorectal cancer (CRC) remains one of the most common malignancies worldwide and a major contributor to cancer-related morbidity and mortality (1). Histopathological examination is central to diagnosis, grading, treatment planning, and quality assurance across the colorectal cancer care pathway. Despite this central role, routine pathology remains labor-intensive and highly dependent on expert interpretation, especially when large numbers of biopsy or resection slides are reviewed under time pressure.

Digital pathology has created an opportunity to develop computational systems that can assist pathologists by screening whole-slide images, flagging suspicious areas, prioritizing cases, and providing quality-control checks (2, 3). Deep learning has become the dominant computational approach because convolutional neural networks, transformer-based models, and related architectures can learn complex image representations directly from digitized histological images. In colorectal pathology, deep learning systems have been applied to cancer detection, tissue segmentation, gland classification, polyp characterization, molecular subtype prediction, prognosis, and treatment-response prediction (4, 5).

However, the apparent progress of AI pathology is difficult to interpret from individual studies alone. Many studies report patch-level or tile-level accuracy, segmentation performance, area under the curve (AUC) values without operating thresholds, public image-based dataset classification results, or molecular/prognostic endpoints rather than clinically interpretable diagnostic contingency tables. Even when diagnostic performance is reported, studies may differ in target condition, unit of analysis, thresholding strategy, model aggregation rule, reference standard, and validation setting.

These issues matter because clinical implementation requires more than a high AUC or visually impressive heatmaps. For a diagnostic adjunct, sensitivity determines how safely the system can avoid missed malignant cases, whereas specificity determines the false-positive workload imposed on pathologists. Patch-level results cannot be assumed to translate into slide-level or patient-level diagnostic performance. Denominator size and endpoint definition can also materially affect crude specificity. Pooled estimates may appear more favorable if large benign cohorts or *post-hoc* dichotomizations are mixed with stricter cancer-detection studies.

Diagnostic test accuracy (DTA) meta-analysis provides a formal framework for addressing these concerns. By requiring extractable or reconstructable true-positive, false-positive, false-negative, and true-negative values, it focuses the review on clinically interpretable operating points rather than on non-thresholded model discrimination alone. The bivariate random-effects model can jointly synthesize sensitivity and specificity while accounting for the negative correlation between them across studies (6, 7). This approach is especially important in AI pathology, where the same broad label of “colorectal cancer detection” can encompass substantially different tasks.

This systematic review and DTA meta-analysis evaluated clinically interpretable slide-level or patient-level diagnostic accuracy of deep learning models for CRC detection on histopathological images, with sensitivity analyses that separated strict primary evidence from endpoint-caveat cohorts.

## Methods

### Study design and reporting framework

This systematic review and diagnostic test accuracy meta-analysis evaluated the performance of deep learning models for detecting colorectal cancer or colorectal adenocarcinoma on histopathological images. The review question was structured according to a diagnostic test accuracy framework: the population/material of interest was human colorectal histopathological tissue, the index test was a deep learning-based image-analysis model, the reference standard was pathological diagnosis or expert pathologist annotation, and the target condition was colorectal cancer, colorectal adenocarcinoma, or a closely aligned malignant colorectal histopathology endpoint. The review was designed and reported in accordance with PRISMA 2020 and PRISMA-DTA principles (8, 9), and risk of bias and applicability were assessed using QUADAS-2 (10). Reporting considerations for diagnostic accuracy studies and AI diagnostic studies were also considered (11, 12). The review protocol was not prospectively registered in PROSPERO; this was treated as a methodological limitation. Because several judgment-dependent decisions (notably the classification of endpoint-caveat cohorts and the rules for accepting reconstructed 2 x 2 tables) evolved as full texts were reviewed, a protocol decision/deviation table distinguishing pre-specified from *post-hoc* decisions is provided in **Supplementary Table 8**.

### Search strategy and information sources

Formal searches were performed in PubMed, Embase, Scopus, Web of Science Core Collection, and IEEE Xplore. The databases were searched from inception to 10 May 2026, with the Web of Science export completed on 11 May 2026. The searches used three concept blocks: colorectal cancer, histopathology or whole-slide/digital pathology imaging, and deep learning or artificial intelligence. No diagnostic-accuracy block or exclusionary NOT block was applied at the search stage because relevant computational pathology studies may contain diagnostic performance data even when the abstract also mentions segmentation, prognosis, molecular prediction, or other related tasks. The exact database-specific strategies are provided in the **Supplementary Material**.

The formal database searches identified 4,687 records: 618 from PubMed, 1,538 from Embase, 1,587 from Scopus, 896 from Web of Science Core Collection, and 48 from IEEE Xplore. Records were exported with available identifiers, title, abstract, journal, year, DOI, and publication metadata. No commercial systematic-review platform (e.g., Covidence, DistillerSR, Rayyan, EndNote, or Zotero) was used; deduplication was performed using custom Python scripts that assigned each record a single matching key, in order of availability—the DOI if present, otherwise the PubMed identifier if present, otherwise the normalized title—and removed subsequent records sharing that key with an already retained record (publication year was not part of the matching key). No fuzzy or similarity-based matching was applied. Subscription-database records (Embase, Scopus, Web of Science, and IEEE Xplore) were deduplicated against one another using this procedure and then reconciled against the locked PubMed screening set using the same matching logic. Overall, 2,380 records were removed before screening because they were duplicates or already represented in the PubMed screening, leaving 2,307 unique records for title/abstract screening. A *post-hoc* audit of the locked screening file identified 62 residual candidate groups (17 sharing a DOI string and 45 sharing a normalized title and publication year) that were not collapsed by the single-key matching procedure described above; each group was individually adjudicated against the source titles. The 17 DOI-sharing groups were found to be unrelated publications linked by erroneous or coincidental DOI metadata, not true duplicates. The 45 title/year-sharing groups were found to be genuine duplicate listings of the same record (chiefly punctuation or formatting differences between database exports, or the same abstract cross-listed in more than one screening queue) that the matching key missed because at least one copy lacked a DOI or PMID. In every one of the 62 groups, all member records were independently excluded at either the title/abstract or detailed-eligibility stage; none contained a record that was included in the final study set, so the eight retained studies, the 158/150 detailed eligibility counts, and the PRISMA flow diagram were unaffected. The 2,380/2,307 counts should accordingly be interpreted as the counts produced by the single-key matching procedure as implemented, not as a fully deduplicated count under a stricter normalized-title match; the full adjudication table is provided in the **Supplementary Material**. The deduplication scripts, the reconciliation log, the duplicate-candidate adjudication record, and the resulting deduplicated record counts are provided in the **Supplementary Material** and reproducibility archive.

As a public-index safety check, Europe PMC, Crossref, and OpenAlex were queried using the same three-concept structure and reconciled against the locked PubMed screen. These public-index searches were used as a supplementary safeguard and were not counted as formal PRISMA database sources.

### Eligibility criteria

Studies were eligible for the primary review if they met all of the following criteria: original research article; human colorectal histopathological material; histology or whole-slide pathology images, primarily hematoxylin and eosin (H&E)-stained images, with other routine staining methods accepted when histological evaluation was equivalent; a deep learning model used as the index test; a diagnostic task focused on colorectal cancer, colorectal adenocarcinoma, or malignant colorectal tissue detection; a pathological diagnosis, expert pathologist label, or study-defined pathology reference standard; and sufficient information to extract or reconstruct a 2 x 2 diagnostic table at slide level or patient level.

Studies were excluded if they were reviews, editorials, comments, protocols, conference abstracts without usable full data, non-human studies, non-histopathology imaging studies, endoscopy- or radiology-only studies, or studies using only non-deep learning methods. Studies were also excluded from the primary quantitative synthesis if they focused only on segmentation, gland or cell detection, molecular subtype prediction, prognosis, treatment response, survival, stain normalization, image retrieval, representation learning, lymph-node metastasis detection, or colorectal polyp subtype classification without an extractable colorectal cancer detection endpoint. Studies reporting only AUC, accuracy, F1 score, patch-level metrics, or qualitative examples without enough information to reconstruct true-positive (TP), false-positive (FP), false-negative (FN), and true-negative (TN) values were not included in the primary meta-analysis. When multiple reports appeared to arise from overlapping cohorts or closely related development programs, only one source was retained in the primary synthesis to avoid double-counting; related reports were documented narratively.

The primary quantitative synthesis was restricted to slide-level or patient-level cancer-detection endpoints that aligned with strict colorectal cancer, colorectal adenocarcinoma, or malignant colorectal histopathology detection. Studies with related but non-identical endpoints were retained only for expanded or sensitivity analyses.

### Study selection and data extraction

Study selection used a two-stage process. Titles and abstracts were first screened against the predefined eligibility criteria. Potentially eligible studies and diagnostically plausible borderline records then underwent a detailed full-text or report-level eligibility assessment. Exclusion reasons were recorded using a structured decision log, with particular attention to the following: wrong target condition, wrong imaging modality, non-diagnostic endpoint, segmentation-only task, molecular or prognostic endpoint, patch- or image-level-only reporting, absence of reconstructable 2 x 2 data, and duplicate or overlapping evidence.

The original systematic review workflow—searching, deduplication, title/abstract screening, full-text eligibility assessment, data extraction, and QUADAS-2 assessment—was conducted primarily by one reviewer (Xiang Yong), with a rule-based script assisting with title/abstract screening. In response to peer review, a second reviewer (Zhen Li) independently verified the original 157 full-text eligibility records and additionally adjudicated the recovered iMIL4PATH report, yielding 158 reports assessed for eligibility in the revised PRISMA accounting. The second reviewer also independently verified the 2 x 2 extractions of included studies for all 21 validation cohorts and all QUADAS-2 judgments across the eight included studies (48 domain assessments), and re-screened all 2,307 unique title/abstract records blind to the original decisions. Title/abstract agreement was 93.5% (prevalence-and-bias-adjusted kappa, 0.87); Cohen’s kappa (0.22) was deflated by the extreme rarity of the advance decision (kappa paradox), so agreement is reported using the observed proportion of agreement and the prevalence-and-bias-adjusted kappa. Of 151 direct disagreements, discordances were resolved through discussion and author adjudication. One eligible study initially excluded by the rule-based screen (iMIL4PATH) was recovered and added to the synthesis, and no eligible study remained unidentified after second-reviewer verification. Independent re-extraction did not change any value used in the strict primary synthesis. A full eligibility adjudication log and the QUADAS-2 worksheet are provided in the **Supplementary Material**. The coauthors critically reviewed the manuscript, the interpretation of the findings, and endpoint-caveat handling.

For each included cohort, the following variables were extracted: first author, publication year, country or region, journal, target condition, image type, staining method, unit of analysis, dataset type, validation design, external validation status, model family, model name, reference standard, threshold or slide/patient aggregation rule, sample size, number of images or cases, TP, FP, FN, TN, reported sensitivity, reported specificity, reported AUC, and extraction notes. When a study reported multiple independent validation cohorts, each cohort was extracted as a separate row.

Data extraction prioritized explicitly reported confusion matrices or exact TP, FP, FN, and TN values. If these were not directly reported, counts were reconstructed only when the published denominators and sensitivity/specificity, class-specific performance table, or figure-level confusion matrix allowed a unique integer 2 x 2 table. Reconstructions from rounded percentages were accepted only when a single integer solution was compatible with the reported denominator and performance estimate. AUC-only reports and non-thresholded performance summaries were not reconstructed.

### Definition of the 2 x 2 table

TP was defined as a slide or patient with the target condition correctly classified as positive by the deep learning system. FN was defined as a slide or patient with the target condition classified as negative. TN was defined as a non-target slide or patient correctly classified as negative, and FP was defined as a non-target slide or patient classified as positive. For multiclass classifiers, the clinically relevant cancer or adenocarcinoma class was dichotomized as test-positive when this dichotomization was supported by the article’s reported decision rule or confusion matrix. For hierarchical or patch-to-slide systems, the published slide-level or patient-level aggregation rule was used. When the positive reference condition included high-grade dysplasia or broader high-risk tissue rather than invasive colorectal cancer, the cohort was excluded from the strict primary analysis but could be retained in expanded or sensitivity analyses.

### Risk of bias and applicability assessment

Risk of bias and applicability were assessed at the source-study level using QUADAS-2 domains: patient selection, index test, reference standard, and flow and timing (10). Each domain was rated as low, high, or unclear risk. Because the review focused on AI pathology models, two additional project-specific considerations were documented: risk of data leakage and external-validation/applicability concern. Data leakage concern considered whether training, validation, and test partitions were separated at an appropriate patient or slide level, particularly when models were trained on patches or tiles. External-validation/applicability concern considered whether the reported validation setting, target condition, threshold, and unit of analysis matched the review question and intended clinical use case.

### Statistical analysis

The primary analysis used all strict endpoint cohorts with extractable slide-level or patient-level TP, FP, FN, and TN data. Sensitivity was calculated as TP/(TP + FN), and specificity as TN/(TN + FP). Crude pooled sensitivity and specificity were calculated by summing TP, FP, FN, and TN across eligible cohorts; however, the main cohort-level synthesis used a bivariate random-effects diagnostic accuracy model. Each validation cohort was treated as an independent observation. This assumption is imperfect when multiple cohorts originate from the same source study because shared model development, thresholding, sampling, or institutional procedures can induce within-study correlation. Consequently, treating such cohorts as independent may underestimate uncertainty and result in overly precise confidence intervals. Model-based heterogeneity estimates were interpreted cautiously because the dataset contained very large denominators, only three source studies, and multiple validation cohorts from the same development program; zero-cell entries also required a continuity correction.

The bivariate model was fitted using the Reitsma approach implemented in the R package mada, with restricted maximum likelihood estimation (6, 13). Model inputs were cohort-level TP, FN, FP, and TN. Because zero cells were present in the extracted 2 x 2 tables, a continuity correction of 0.5 was applied to all cohorts according to the mada::reitsma() default rule (correction = 0.5, correction.control = “all”). The model jointly synthesizes sensitivity and the false-positive rate on transformed scales, allowing for the correlation between sensitivity and specificity across validation cohorts. Summary sensitivity, summary false-positive rate, derived summary specificity, 95% confidence intervals, and summary receiver operating characteristic estimates were extracted from the fitted model. Forest plots of sensitivity and specificity and summary receiver operating characteristic (SROC) plots were generated for the primary strict endpoint dataset and for the expanded all-evaluable dataset. Analyses were conducted in R (14).

Sensitivity analyses were prespecified to examine the influence of endpoint definition, validation design, and unit of analysis, and were supplemented by additional robustness analyses requested during peer review. The scenarios evaluated were: primary strict endpoint cohorts only; all extracted evaluable cohorts, including endpoint-caveat cohorts; endpoint-caveat cohorts only; exclusion of the *post-hoc* Ko 2022 quality-control cohort; primary strict and expanded external-validation cohorts only; exclusion of the dominant Wang 2021 source study; exclusion of cohorts with indirectly derived (reconstructed) 2 x 2 tables; exclusion of cohorts rated at high overall risk of bias; and a leave-one-study-out analysis removing each source study in turn. Scenarios that retained fewer than two cohorts were reported descriptively only and were not treated as reliable bivariate syntheses. Source-clustered inference was also considered in response to the editor. However, the strict primary dataset contained only three source studies, with cluster sizes of 12, 1, and 1 cohorts. Cluster-robust inference with three clusters is unreliable, and aggregation to the source-study level would leave only three observations for a five-parameter bivariate random-effects model. We therefore report descriptive source-level 2 x 2 summaries and the leave-Wang-out sensitivity analysis while treating all cohort-level pooled estimates as exploratory.

## Results

### Study selection

**Figure 1:**
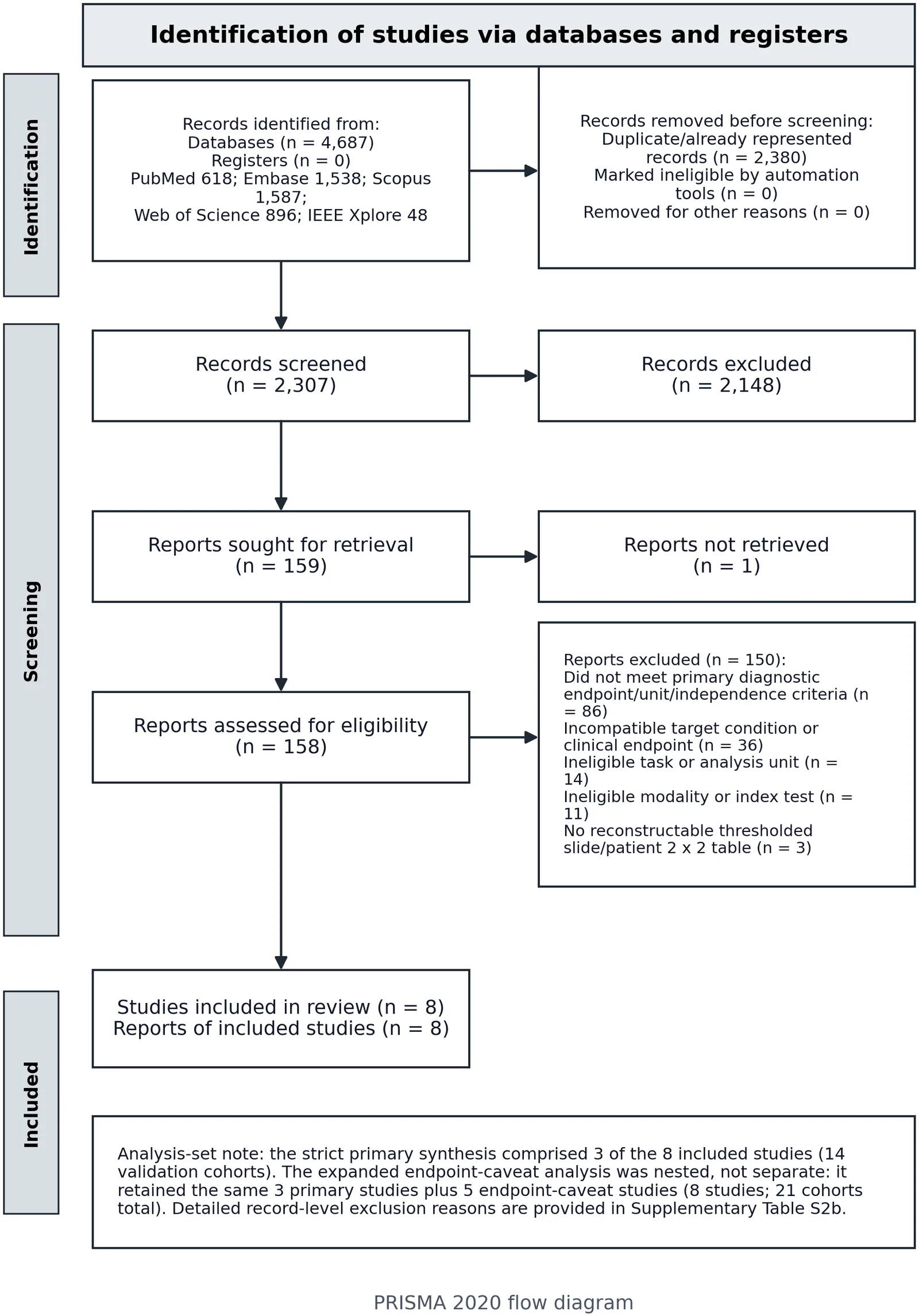
PRISMA 2020 flow diagram for the formal multi-database search. Formal databases were PubMed, Embase, Scopus, Web of Science Core Collection, and IEEE Xplore. Europe PMC, Crossref, and OpenAlex were supplementary safety checks and were not counted as formal PRISMA database sources. The final “Included” box reports all eight included studies. The strict primary synthesis comprised three of these studies (14 cohorts); the expanded endpoint-caveat analysis was nested and retained the same three primary studies plus five endpoint-caveat studies (eight studies and 21 cohorts total). Detailed record-level exclusion reasons are provided in **Supplementary Table 2b**.

The formal multi-database search identified 4,687 records: 618 from PubMed, 1,538 from Embase, 1,587 from Scopus, 896 from Web of Science Core Collection, and 48 from IEEE Xplore. After the removal of within-subscription duplicates and records already represented in the locked PubMed screen, 2,380 records were removed before screening, and 2,307 unique records underwent title/abstract screening. At the title and abstract level, 2,148 records were excluded; one eligible report (iMIL4PATH) that had been excluded by the rule-based title/abstract screen was recovered during independent second-reviewer re-screening and moved into the eligibility process. A total of 159 reports entered the retrieval or detailed eligibility process; one report was not retrieved, and 158 reports were assessed for eligibility. Of these, 150 were excluded after detailed assessment. Three source studies, contributing 14 validation cohorts, met the strict endpoint criteria for the primary diagnostic accuracy synthesis. Five additional source studies, contributing seven validation cohorts, were retained in an expanded all-evaluable analysis because their endpoints were adjacent to, but not fully aligned with, strict CRC detection (**Figure 1**).

The 1,689 additional records from non-PubMed databases did not identify any additional studies with extractable slide-level or patient-level TP, FP, FN, and TN data for the strict primary meta-analysis. The most frequent reasons for exclusion or non-inclusion were a mismatch between the unit of analysis and the review question, a wrong imaging modality or non-routine histopathology modality, segmentation or cell-level tasks, prognosis or treatment-response endpoints, molecular or biomarker-prediction endpoints, polyp or adenoma classification, lymph-node metastasis detection, and review or commentary articles.

### Study characteristics

The primary synthesis included three source studies evaluating deep learning systems on hematoxylin and eosin-stained colorectal histopathology whole-slide images (15–17). The 14 primary validation cohorts comprised 13 patient-level cohorts and one slide-level cohort. Primary cohorts represented China and the United States. The majority of cohorts used private datasets; one cohort used a public dataset. A total of 11 primary cohorts were marked as externally validated, while three used internal or non-external validation settings. The expanded all-evaluable analysis included five additional source studies from the Republic of Korea, Germany and Austria, Italy, Ireland and the United Kingdom, and Portugal, bringing the all-evaluable dataset to eight source studies and 21 validation cohorts (18–22).

The primary target conditions included colorectal cancer versus non-colorectal cancer, adenocarcinoma versus non-adenocarcinoma, and malignant or colorectal cancer-containing slides versus benign slides. Wang (2021) evaluated colorectal cancer versus non-colorectal cancer at the patient level (15). Kim (2023) evaluated adenocarcinoma versus non-adenocarcinoma in colorectal resection whole-slide images (16). Yuan (2024) evaluated malignant or colorectal cancer-containing slides versus benign slides in an external validation cohort (17). Ko (2022), Griem (2023), Bokhorst (2023), White (2025), and iMIL4PATH (Neto, 2022) were excluded from the primary synthesis because of endpoint caveats (18–22). Ko (2022) contributed a large slide-level clinical quality-control dataset, but the strict carcinoma-versus-non-carcinoma extraction was reconstructed *post hoc* from a broader gastrointestinal biopsy quality-control classifier rather than representing a standard prospective CRC-versus-non-CRC diagnostic endpoint. Griem (2023) contributed two slide-level validation cohorts for tumor or high-grade dysplasia versus benign tissue and therefore did not represent a pure invasive CRC-versus-non-CRC endpoint. Bokhorst (2023) contributed a slide-level colorectal biopsy cohort whose target condition grouped cancer with other clinically relevant abnormalities rather than isolating invasive CRC; its 2 x 2 table was reconstructed from the published slide-level confusion matrix and retained only as endpoint-caveat evidence. White (2025) contributed two slide-level case-prioritization cohorts (one internal and one external) in which the positive endpoint combined high-grade dysplasia and cancer (one-versus-rest) rather than isolating invasive CRC. iMIL4PATH (Neto, 2022) contributed one slide-level cohort from a multiple-instance-learning colorectal biopsy classifier whose positive class extended beyond strict invasive carcinoma and carried higher data-leakage and index-test uncertainty; it was recovered during independent second-reviewer re-screening and retained only as endpoint-caveat evidence.

### Diagnostic accuracy

**Figure 2:**
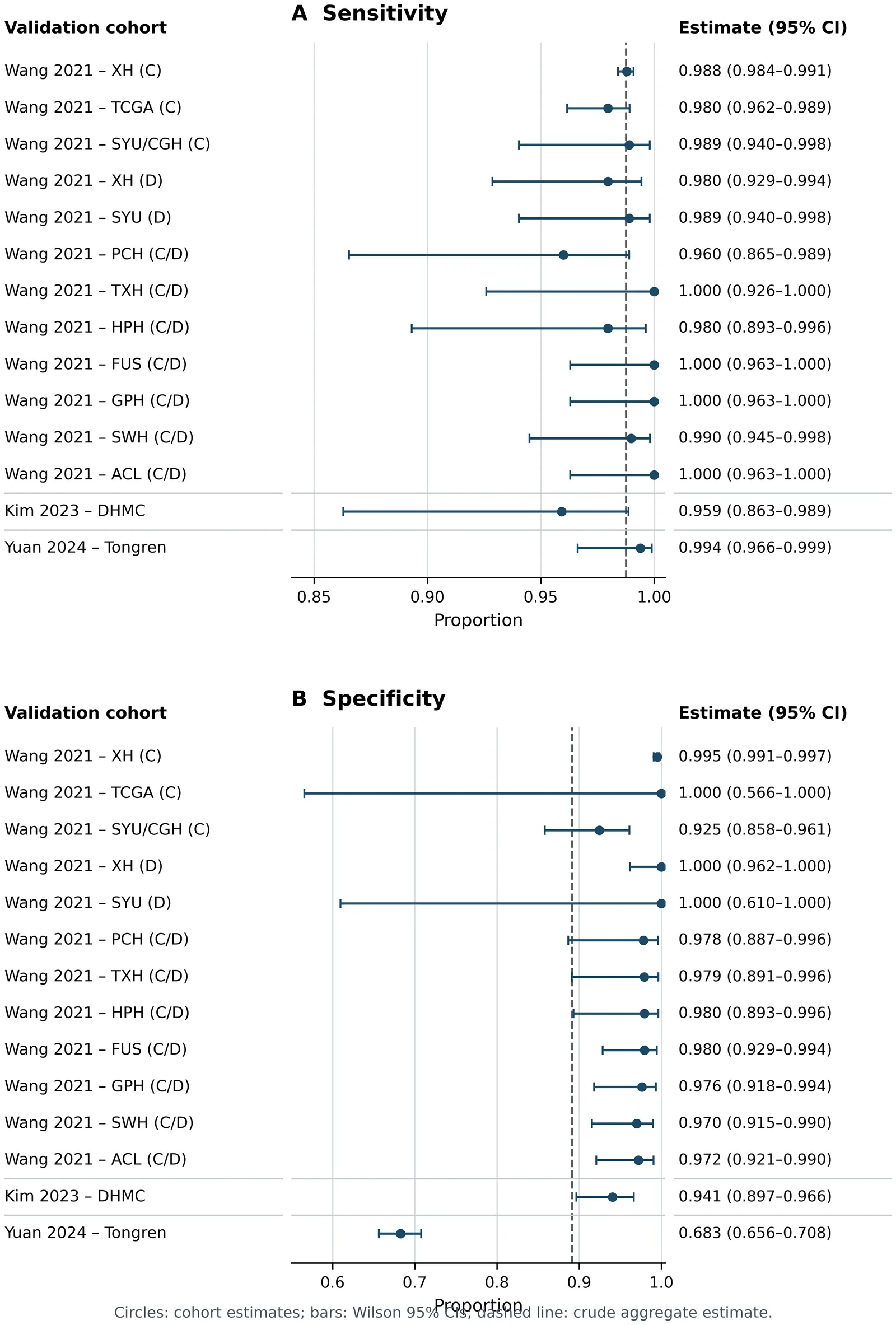
Forest plots for sensitivity **(A)** and specificity **(B)** in the primary strict endpoint dataset, reformatted as stacked panels to preserve legibility at publication size. Circles show cohort-level estimates, horizontal bars show Wilson 95% confidence intervals, and the right column reports the numerical estimate and interval. The dashed vertical line indicates the crude estimate from the summed 2 x 2 counts. Yuan 2024 is the only slide-level cohort in the strict primary synthesis; the remaining primary cohorts are patient-level cohorts.

Across the 14 primary validation cohorts, the extracted 2 x 2 tables included 5,402 true positives, 439 false positives, 68 false negatives, and 3,596 true negatives. The crude pooled sensitivity across primary rows was 0.988, and the crude pooled specificity was 0.891 (**Figure 2**).

**Figure 3:**
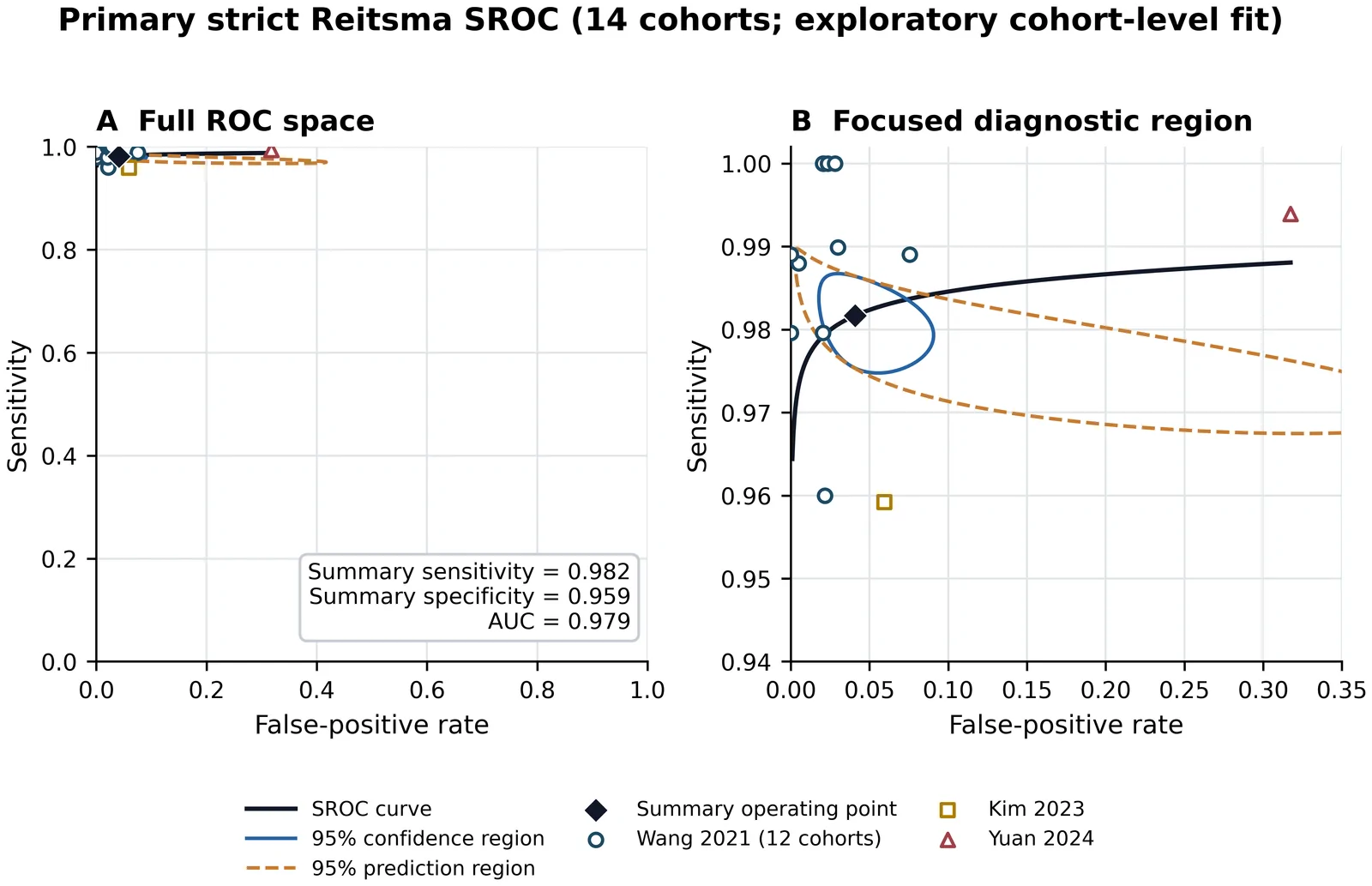
Summary receiver operating characteristic plot for the primary strict endpoint dataset fitted using the Reitsma bivariate random-effects diagnostic accuracy model. **(A)** shows the full 0–1 ROC space; **(B)** magnifies the observed diagnostic region. Cohort-level estimates, the fitted SROC curve, summary operating point, 95% confidence region, and 95% prediction region are shown. The summary AUC was 0.979. Because only three source studies contributed to the model and the estimated random-effects correlation was at the boundary (−1.000), the pooled point, confidence region, and prediction region are exploratory and should be interpreted cautiously.

In the primary bivariate random-effects diagnostic accuracy model, the exploratory summary sensitivity was 0.982 (95% CI, 0.976–0.986). The summary false-positive rate was 0.041 (95% CI, 0.021–0.078), corresponding to a derived summary specificity of 0.959 (95% CI, 0.922–0.979). The summary AUC was 0.979, with a normalized partial AUC of 0.975 (**Figure 3**). These are cohort-level model estimates, not source-clustered estimates. Because 12 of the 14 cohorts originated from Wang (2021), the independence assumption may have resulted in overly precise confidence intervals if within-study cohorts were correlated. On the logit scale used by the bivariate model, the between-cohort standard deviations were 0.200 for logit sensitivity and 1.099 for logit false-positive rate, with an estimated between-cohort correlation of −1.000 between the two random effects. This correlation at the boundary of the parameter space, rather than reflecting a genuinely perfect inverse relationship, indicates that the variance-covariance structure is weakly identified given only 14 cohorts from three source studies, and that model-based heterogeneity parameters from this fit should be interpreted as imprecise. Heterogeneity was also quantified using I² estimates appropriate for bivariate diagnostic accuracy data. The Zhou–Dendukuri approach gave I² = 0%, the sample-size-unadjusted Holling approach gave I² = 28.5%–62%, and the sample-size-adjusted Holling approach gave I² = 0.4%–0.7%. This wide range across estimators, combined with the boundary correlation estimate, indicates that heterogeneity in this dataset cannot be summarized by a single reliable figure. Estimates should therefore be interpreted cautiously because the synthesis included only three primary source studies and multiple cohorts from one development program, and required continuity corrections for zero-cell entries, which may have further affected both the variance-covariance estimates and the I² calculations.

A descriptive source-study-level analysis is provided in **Table 1**. Wang (2021) contributed 12 validation cohorts and accounted for the majority of the patient-level evidence; its aggregated crude sensitivity and specificity were both 0.988. Kim (2023) contributed one patient-level internal test cohort, with crude sensitivity of 0.959 and specificity of 0.941. Yuan (2024) contributed one slide-level external validation cohort, with crude sensitivity of 0.994 and specificity of 0.683. These source-level values describe the three evidence sources but are not an alternative pooled bivariate estimate.

**Table 1:** Source-study-level summary of the eight studies retained in the quantitative or sensitivity dataset.

Study	Analysis	Cohorts	Unit	Target/validation	TP/FP/FN/TN	Sens/spec (%)
Wang, 2021 (15)	Strict primary	12	Patient	CRC vs non-CRC; 10 external	5192/30/65/2566	98.8/98.8
Kim, 2023 (16)	Strict primary	1	Patient	ADC vs non-ADC; internal test	47/11/2/174	95.9/94.1
Yuan, 2024 (17)	Strict primary	1	Slide	Malignant/CRC vs benign; external	163/398/1/856	99.4/68.3
Ko, 2022 (18)	Endpoint caveat	1	Slide	QC carcinoma reconstruction	50/185/1/12213	98.0/98.5
Griem, 2023 (19)	Endpoint caveat	2	Slide	Tumor/HGD vs benign; 1 external	194/19/0/354	100.0/94.9
Bokhorst, 2023 (21)	Endpoint caveat	1	Slide	Abnormality (including cancer); internal	265/14/27/748	90.8/98.2
White, 2025 (20)	Endpoint caveat	2	Slide	HGD/cancer vs rest; 1 external	31/2/3/165	91.2/98.8
iMIL4PATH	Endpoint caveat	1	Slide	Broader positive class; internal	48/2/14/195	77.4/99.0

Complete cohort-level characteristics for all 21 validation cohorts are provided in **Supplementary Table 3**.

TP, true positive; FP, false positive; FN, false negative; TN, true negative; Sens, sensitivity; Spec, specificity; CRC, colorectal cancer; ADC, adenocarcinoma; HGD, high-grade dysplasia; QC, quality control. Counts and crude sensitivity and specificity are descriptive values aggregated within each source study; they are not source-clustered pooled estimates. The strict primary cohort-level Reitsma model treated cohorts as independent, but reliable cluster-robust or source-study-level bivariate inference was not feasible with only three strict primary source studies. Full cohort-level details, reference standards, thresholds and aggregation rules, extraction methods, and AUC values are provided in **Supplementary Table 3**.

### Sensitivity analyses

Sensitivity analyses showed that crude sensitivity remained stable across alternative analytic scenarios, whereas specificity was more sensitive to study selection, endpoint definition, and unit of analysis. In the primary strict endpoint dataset, crude sensitivity was 0.988, and crude specificity was 0.891. In the expanded all-evaluable dataset, which added the five endpoint-caveat studies (seven cohorts), crude sensitivity was 0.981, and crude specificity rose to 0.963, reflecting the large benign (true-negative) denominators contributed by the caveat cohorts. Removing the *post-hoc* quality-control extraction from Ko (2022) from the expanded dataset preserved crude sensitivity at 0.981 but reduced crude specificity to 0.914, illustrating the strong influence of that single very large true-negative cohort on the crude estimate.

The expanded all-evaluable bivariate model, which retained the endpoint-caveat cohorts, produced a summary sensitivity of 0.974 (95% CI, 0.954–0.985) and a derived specificity of 0.967 (95% CI, 0.946–0.980), with an AUC of 0.987 and a normalized partial AUC of 0.981. Thus, the cohort-level point estimates and partial AUC were numerically similar across the strict and expanded datasets, whereas crude specificity was strongly affected by large true-negative denominators. Restricting the primary strict dataset to external-validation cohorts yielded a crude sensitivity of 0.988 and a specificity of 0.780 across 11 cohorts, with a model-based specificity of 0.947 (95% CI, 0.899–0.973); this lower crude specificity was mainly attributable to the external slide-level Yuan (2024) cohort. The corresponding expanded external-validation dataset (13 cohorts) yielded a crude sensitivity of 0.987 and a specificity of 0.796, with a model-based sensitivity of 0.976 and specificity of 0.953. In the source-dominance sensitivity analysis, excluding Wang (2021) from the strict dataset left only Kim (2023) and Yuan (2024) (two source studies and two cohorts), with a crude sensitivity of 0.986 and a specificity of 0.716. A cohort-level Reitsma fit was computationally obtainable for these two observations (sensitivity, 0.984; specificity, 0.851), but its specificity confidence interval was extremely wide (0.447–0.976); it was therefore treated as unstable descriptive evidence rather than as a valid source-clustered pooled estimate. Excluding Wang (2021) from the expanded dataset left nine cohorts from seven source studies and produced a summary sensitivity of 0.942 and a specificity of 0.966 (AUC, 0.981). Leave-one-study-out removal of each of the other seven source studies changed the expanded summary sensitivity and specificity by no more than approximately 0.01. After excluding cohorts with indirectly derived 2 x 2 tables or a high overall risk of bias, the strict dataset retained only a single cohort (Yuan, 2024) and could not be synthesized; the corresponding expanded-dataset analyses remained estimable (summary sensitivity, approximately 0.94; specificity, approximately 0.96). Full per-scenario bivariate model estimates, leave-one-study-out fits, and heterogeneity components are provided in **Supplementary Table 5**.

### Endpoint definition and near-miss evidence

Several closely related studies were deliberately not pooled in either the strict or expanded synthesis because their target condition, reporting format, or independence from the included evidence did not match the review’s inclusion criteria. Yu (2021) was treated as potentially overlapping evidence from a closely related multicenter colorectal WSI program and was therefore not pooled as an independent source study (23). Ho (2022) addressed a broader high-risk versus low-risk biopsy triage task rather than a strict invasive colorectal carcinoma endpoint (24). Bilal (2023) reported a composite neoplasia endpoint (low-grade adenomas, serrated lesions, and carcinoma grouped as a single positive class) that was broader than, and not compatible with, the strict or endpoint-caveat CRC-detection definitions used here and was therefore excluded (25). Bilal (2022), a medRxiv preprint from the same report family that has not undergone peer review, evaluated AI-based pre-screening of large-bowel biopsy histology with a broader neoplastic-versus-normal endpoint and did not provide an exact thresholded slide-level or patient-level 2 x 2 table suitable for strict invasive CRC detection; it was identified outside the formal database search, used only as background near-miss evidence, and not included in the quantitative synthesis (26). Schrammen (2022) provided weakly supervised digital pathology evidence but did not report a directly extractable thresholded slide-level or patient-level CRC 2 x 2 table (27). Xu (2023) was clinically adjacent but was not pooled because its primary report did not provide a directly usable strict slide-level or patient-level CRC diagnostic 2 x 2 table for the primary model (28). By contrast, Bokhorst (2023), White (2025), and iMIL4PATH (Neto, 2022), which had been treated as near-miss evidence in the original submission, were reclassified as endpoint-caveat cohorts and added to the expanded all-evaluable synthesis after independent second-reviewer adjudication yielded reconstructable slide-level 2 x 2 tables aligned with the tumor/high-grade endpoint-caveat rule (20–22).

### Risk of bias and applicability

Risk of bias assessment identified recurrent concerns in patient selection and index-test specification (**Table 2**). Patient selection risk was rated high in two studies (Wang, 2021 and Kim, 2023), unclear in five studies, and low in one study (Yuan, 2024). Index-test risk was rated high in three studies (Wang, 2021; Ko, 2022; and iMIL4PATH) and low in the remaining five, mainly because threshold selection, aggregation rules, or *post-hoc* dichotomization were not always specified within a conventional diagnostic-test-accuracy framework. Reference-standard risk was lower overall, with seven studies rated low and one rated high (Ko, 2022). Flow and timing were rated low in all eight studies.

**Table 2:** Source-study-level QUADAS-2 summary.

Study	Patient selection	Index test	Reference standard	Flow and timing	Data leakage	Applicability concern
Wang, 2021 (15)	High	High	Low	Low	Unclear	Low
Kim, 2023 (16)	High	Low	Low	Low	Low	Unclear
Yuan, 2024 (17)	Low	Low	Low	Low	Low	Low
Ko, 2022 (18)	Unclear	High	High	Low	Low	High
Griem, 2023 (19)	Unclear	Low	Low	Low	Low	High
Bokhorst, 2023 (21)	Unclear	Low	Low	Low	Unclear	High
White, 2025 (20)	Unclear	Low	Low	Low	Unclear	Unclear
iMIL4PATH	Unclear	High	Low	Low	High	High

Ratings represent source-study-level summary judgments. Per-domain signaling-question guides, human ratings, exact evidence locations, and the independent reviewer’s rationale for all eight studies (48 domain assessments) are provided in **Supplementary Table 4**.

Applicability concerns were driven mainly by endpoint heterogeneity, differences between patient-level and slide-level analysis, *post-hoc* reconstruction of binary endpoints, and limited external validation in some studies. Applicability concern was rated high in four studies (Ko, 2022; Griem, 2023; Bokhorst, 2023, and iMIL4PATH), unclear in two studies (Kim, 2023, reflecting its retrospective single-center internal held-out test set and the absence of a fully external binary diagnostic validation cohort; and White 2025, because its internal MMUH validation cohort was from the same institution, whereas the NHS cohort was fully external), and low in two studies (Wang, 2021 and Yuan, 2024). Data-leakage risk was rated low in four studies, unclear in three (Wang, 2021; Bokhorst, 2023; and White, 2025), and high in one (iMIL4PATH), reflecting the common use of patch- or tile-level training with higher-level slide or patient aggregation and incomplete reporting of partitioning safeguards in some articles.

## Discussion

### Principal findings

In the primary strict endpoint analysis, the exploratory cohort-level model estimated a summary sensitivity of 0.982 and a derived specificity of 0.959, with a summary AUC of 0.979. These results suggest strong discrimination within the available validation cohorts, but they do not establish a mature, independently replicated, multisource evidence base because the 14 cohorts arose from only three source studies, and 12 were contributed by Wang (2021).

The apparent strength and precision of the pooled performance should therefore be interpreted carefully. Validation cohorts from the same source study may be correlated through shared model development, thresholding, sampling, or institutional procedures. Treating them as independent can produce confidence intervals that are too narrow, and the current three-source dataset was insufficient for reliable source-clustered inference. In addition, the included studies were not fully homogeneous with respect to target condition, unit of analysis, validation design, or thresholding strategy. The most robust evidence appears to support patient-level colorectal cancer versus non-colorectal cancer classification, while slide-level and broader biopsy-triage settings showed greater variability, especially in specificity.

### Interpretation of sensitivity and specificity

The consistently high sensitivity across analytic scenarios is clinically encouraging. For screening, prescreening, or triage applications, a high-sensitivity system may help reduce the risk of missed malignant cases and assist pathologists by flagging slides or cases that require closer review. The low number of false-negatives results across the extracted validation cohorts supports the potential value of deep learning systems as a safety-oriented adjunct in digital pathology workflows.

Specificity was less stable. This was particularly evident in slide-level external validation, where false-positive calls were more frequent than in patient-level cohorts. The lower specificity in Yuan (2024) should be interpreted as a slide-level false-positive burden rather than a patient-level misclassification burden; these units are not clinically interchangeable. Specificity instability was also evident in the sensitivity analyses: adding the five endpoint-caveat studies to the strict primary dataset increased crude specificity from 0.891 to 0.963, and removing Ko (2022) from the expanded dataset reduced crude specificity from 0.963 to 0.914. This demonstrates that crude specificity was strongly influenced by large true-negative denominators, especially in the *post-hoc* Ko (2022) quality-control cohort. The Ko (2022) cohort was informative as a workflow-adjacent sensitivity scenario, but not as co-equal primary CRC detection evidence because its *post-hoc* endpoint reconstruction and very large benign denominator materially altered crude specificity. In contrast, the bivariate model-based specificity was numerically similar between the primary strict analysis and the expanded all-evaluable analysis, suggesting that the central model-based estimate was less affected than the crude denominator-weighted estimate.

### Clinical implications

The current evidence supports deep learning as a promising adjunct for colorectal histopathology rather than as a standalone replacement for pathologist diagnosis. The most immediate clinical use cases are likely to be prescreening, workload prioritization, quality control, and decision support. In these roles, high sensitivity is valuable because the system can help identify slides or cases that warrant prompt attention from a pathologist.

At the same time, variable specificity means that workflow impact cannot be inferred from sensitivity alone. A model with excellent sensitivity but modest specificity may still increase a pathologist’s workload if it generates many false-positive alerts. Therefore, local validation should include not only diagnostic accuracy but also the number of additional slides or regions requiring review, diagnostic turnaround time, false-positive burden, and the clinical consequences of any missed malignant cases.

### Strengths

This review applied a strict diagnostic-accuracy extraction strategy focused on slide-level or patient-level 2 x 2 data. This is important because many colorectal computational pathology studies report patch-level accuracy, tissue segmentation performance, AUC values without thresholded counts, molecular biomarker prediction, prognosis, or public image-based dataset classification. Such studies may be valuable for method development, but do not directly answer the clinical diagnostic accuracy question addressed here.

The review also explicitly separated primary eligible evidence from secondary or edge evidence. Several near-miss studies were retained in the screening log but not pooled, including studies of high-risk biopsy triage, colorectal polyp classification, lymph-node metastasis detection, molecular prediction, segmentation, and image-level public datasets. This conservative approach reduces the risk of inflating diagnostic performance by mixing incompatible endpoints or units of analysis.

Within the locked cohort-level sensitivity scenarios, the strict and expanded models produced numerically similar AUC estimates (0.979 and 0.987), and the normalized partial AUC changed from 0.975 to 0.981 after endpoint-caveat cohorts were added. This numerical stability supports the direction of the discrimination signal within the available data, but it does not correct the dependence among cohorts from Wang (2021) and should not be interpreted as an independent multisource replication.

### Limitations

Several limitations should be considered when interpreting these findings. First, the evidence base remains narrow. Although 14 validation cohorts were included in the primary synthesis, these arose from only three source studies. In addition, Wang (2021) contributed 12 cohorts. Multiple cohorts from the same source study may be correlated; treating them as independent can underestimate standard errors and make confidence intervals overly precise. With only three source studies, neither cluster-robust inference nor a source-study-level five-parameter bivariate random-effects model was statistically reliable. Accordingly, the primary pooled estimates are exploratory and represent evidence dominated by one development program rather than evenly distributed multisource replication. Second, endpoint definitions varied across the broader all-evaluable evidence base, including colorectal cancer versus non-colorectal cancer, adenocarcinoma versus non-adenocarcinoma, malignant tissue versus benign tissue, strict carcinoma reconstructed from a quality-control classifier, and tumor or high-grade dysplasia versus benign tissue. Ko (2022), Griem (2023), Bokhorst (2023), White (2025), and iMIL4PATH are the endpoint-caveat studies and were excluded from the primary synthesis.

Third, some 2 x 2 tables required reconstruction from published tables, reported sensitivity and specificity, figures, or *post-hoc* dichotomization. Although these reconstructions were documented, they are less robust than prospectively reported diagnostic contingency tables. Fourth, patient-level and slide-level cohorts were analyzed together in the main exploratory synthesis, even though these units possess different clinical meanings and false-positive mechanisms. Fifth, the majority of included systems were CNN-family models. More recent transformer- and foundation-model studies were generally excluded because they focused on molecular prediction, prognosis, segmentation, public image datasets, or did not report extractable primary diagnostic 2 x 2 data. Sixth, heterogeneity quantification was itself unstable. The bivariate model estimated a between-cohort correlation of −1.000 between logit sensitivity and logit false-positive rate, a boundary value indicating that the variance-covariance structure is weakly identified with only 14 cohorts from three source studies. I² estimates ranged from 0% (Zhou–Dendukuri) to 28.5%–62% (sample-size-unadjusted Holling) to 0.4%–0.7% (sample-size-adjusted Holling). This discordance across estimators, together with the boundary correlation estimate, is an expected consequence of a small number of source studies, large and uneven cohort denominators, and continuity-corrected zero cells. It means that heterogeneity and prediction regions from this fit should not be interpreted as stable precision estimates.

Finally, although the PRISMA flow reflects a formal multi-database search, the quantitative evidence base remains narrow. The additional Embase, Scopus, Web of Science, and IEEE Xplore records improved search completeness and PRISMA credibility, but they did not identify additional studies with extractable slide-level or patient-level diagnostic 2 x 2 data beyond the already retained primary and endpoint-caveat cohorts. The review was not prospectively registered, and screening and extraction were performed primarily by a single reviewer at the time of initial submission, which increases the risk of selection and extraction error. In response to peer review, a second reviewer independently verified all 158 reports assessed for eligibility, the 2 x 2 extractions for the 21 validation cohorts, and all QUADAS-2 judgments, and re-screened all 2,307 title/abstract records blind to the original decisions (title/abstract agreement, 93.5%; prevalence-and-bias-adjusted kappa, 0.87). This verification recovered one previously missed eligible study (iMIL4PATH) and did not change any value used in the strict primary synthesis. Prospective dual-reviewer screening from the outset nonetheless remains preferable.

### Future research

Future diagnostic accuracy studies should use prospective or temporally external validation, prespecified thresholds, and complete reporting of 2 x 2 tables at both slide and patient levels. Studies should clearly distinguish invasive carcinoma, high-grade dysplasia, low-grade dysplasia, benign lesions, normal tissue, and other clinically relevant categories. This would allow future reviews to compare strict cancer-detection performance separately from broader high-risk biopsy-triage performance.

Research should also move beyond model discrimination alone. For clinical implementation, investigators should quantify workflow impact, including workload reduction, case-prioritization accuracy, time savings, false-positive review burden, and the consequences of false-negative cases. Transparent reporting of data partitioning, patch-to-slide aggregation, threshold selection, scanner variation, and external validation is essential for judging generalizability and reducing the risk of optimistic performance estimates.

## Conclusion

This exploratory synthesis suggests that deep learning systems can achieve high sensitivity and strong discrimination for colorectal cancer detection in histopathological whole-slide images. However, the primary evidence comprises only three source studies and is dominated by 12 cohorts from Wang (2021). The narrow cohort-level confidence intervals may therefore overstate precision and should not be interpreted as a mature, independently replicated, multisource evidence base. Specificity remains more variable in slide-level and broader triage settings. Current evidence most strongly supports deep learning as an adjunctive tool for prescreening, triage, and quality-control workflows rather than as a standalone diagnostic replacement.

## Data Availability

All data supporting the findings of this systematic review and meta-analysis are included in the article and its Supplementary Material. Further inquiries can be directed to the corresponding authors.

## Supplementary material

Supplementary Figure 1 Forest plots for sensitivity **(A)** and specificity **(B)** in the expanded all-evaluable dataset, including endpoint-caveat cohorts retained for supportive analyses. Ko 2022 materially increased the crude specificity estimate because of its very large true-negative denominator.

Supplementary Figure 2 Summary receiver operating characteristic plot for the expanded all-evaluable dataset fitted using the Reitsma bivariate random-effects diagnostic accuracy model. Axes are shown on the full 0–1 scale to preserve diagnostic-accuracy interpretability.
